# From Inflammation to Prostate Cancer: The Role of Inflammasomes

**DOI:** 10.1155/2016/3140372

**Published:** 2016-06-27

**Authors:** Dev Karan, Seema Dubey

**Affiliations:** Department of Pathology, Microbiology and Immunology, University of South Carolina School of Medicine, Columbia, SC 29208, USA

## Abstract

Inflammation-associated studies entice specific attention due to inflammation's role in multiple stages of prostate cancer development. However, mechanistic regulation of inflammation inciting prostate cancer remains largely uncharacterized. A focused class of inflammatory regulators known as inflammasomes has recently gained attention in cancer development. Inflammasomes are a multiprotein complex that drives a cascade of proinflammatory cytokines regulating various cellular activities. Inflammasomes activation is linked with infection, stress, or danger signals, which are common events within the prostate gland. In this study, we review the potential of inflammasomes in understanding the role of inflammation in prostate cancer.

## 1. Introduction

Prostate cancer is the most common cancer and the second leading cause of cancer-related deaths among men in the United States [1]. The initiation of tumor in the prostate has been linked with multiple factors including age, race, diet, heredity, and environment [2]. Additionally, inflammation also remains an integral component in prostatic diseases and may contribute to tipping the balance towards tumor cell growth. Over the years, there has been a surge in understanding the role of inflammation in prostate cancer. Based on the presence of infiltrating lymphocytes in the prostate biopsy, it is suggested that the quantity of immune cells infiltration may help to predict the outcome of prostate cancer. However, several other studies are not in consensus with such correlative outcome. In the context of inflammation, the studies remain minimal on the role of inflammatory cytokines, which are potentially regulated via the inflammasome complex [3, 4]. Therefore, we discuss the prospective of inflammasome complex regulating inflammation in the context with prostate cancer.

## 2. Prostate and Inflammation

The common disorders of the prostate include benign prostatic hyperplasia (BPH), prostatitis (chronic inflammation), and prostate cancer. As the prostate gland is compartmentalized into three distinct zones (a central zone (CZ), a transitional zone (TZ), and a peripheral zone (PZ)), it is a general consensus that the BPH develops in the site of TZ whereas prostatitis and cancer mainly occur in the PZ. The CZ of the prostate is rarely involved with carcinoma [5]. The dominant pathological symptoms of BPH and prostate cancer are characterized by extensive proliferation of the prostate glandular cells, while prostatitis is mainly an inflammation-associated disease. The disorders in the prostate primarily progress with the advancement of age, which further increases the susceptibility of the prostate tissue to injury or infection leading to inflammatory response [6–8]. Inflammation has been associated with various cancer types with an estimated 20% of cancer deaths linked to chronic infections and persistence inflammation [9–11]. Inflammation may incite carcinogenesis by causing DNA damage (genetic and epigenetic modulations), promoting cellular proliferation as well as angiogenesis [12–14].

The role of inflammation in prostate cancer has been appreciated for a long time. Classically, inflammation is described based on the physical presence of immune infiltrating cells as a natural response at the site of tissue injury or infection. Although infiltrating immune cells are detected in prostate cancer specimen, the significance of immune cells infiltration (coexistence with prostate tumor) and correlative association (protective or tumor promoting) remains contradictory. Based on histopathologic analysis of prostate tumor biopsy, several studies revealed an inverse association of inflammatory infiltrates with prostate cancer. Retrospective analysis of the prostate biopsies from the REDUCE (Reduction by Dutasteride of Prostate Cancer Events) study demonstrated that inflammation in the negative biopsy for prostate cancer lowers the subsequent prostate cancer detection and reduced risk of prostate cancer in association with baseline inflammation [15, 16]. In a Finnish prostate cancer screening trial, histologic inflammation in prostate biopsy among men with initially elevated serum prostate-specific antigen (PSA) was not linked to increased risk of prostate cancer [17]. Additionally, histological inflammatory finding at the initial prostate biopsy was negatively associated with prostate cancer detection in repeat biopsy [18]. When we consider the aggressiveness of prostate cancer among different ethnicities, African American (AA) men are at a higher risk and carry an aggressive load of disease. However, clinical prostatitis indicated an inverse association with prostate cancer in AA men, and histologic inflammation was not related to prostate cancer disparity [19, 20]. On the contrary, chronic inflammation in the areas of benign prostatic tissue is linked with high-grade prostate cancer. Histologic analysis of prostate biopsy from PCPT (Prostate Cancer Prevention Trial) showed an association of the high-grade disease with inflammation [21]. Overall, the diagnosis of prostate cancer using sampling biopsies collected via transrectal ultrasound guidance seems apprehensive with peril as 25% or more of men with “negative” biopsies show prostate cancer on a subsequent biopsy within months to a year. A recent review summarizes the infiltration of T cells (CD4+, CD8+, and Th17), B cells, macrophages, mast cells, immunosuppressive cells (T regulatory cells and myeloid derived suppressor cells), and neutrophils in association with prostate cancer studies [22]. The infiltration of macrophages and immune suppressor cells was found to be positively associated with prostate cancer progression; however, the coexistence of other cell types with prostate tumors remains inconclusive. Thus, the physical presence of the type and the amount of immune infiltrating cells within the prostate tissue may vary significantly. Indeed, impact of inflammation may be of influence at the individual level such as personal lifestyle including eating habits (diet). Inflammatory infiltrates may also depend on the severity of the injured/damaged tissue. Nonetheless, these infiltrating lymphocytes along with the host tissue secrete a plethora of cytokines/chemokines, which recruit such immune infiltrates and can easily dictate cellular activities towards neoplasm. The legacy of cytokines/chemokines these infiltrating lymphocytes leave behind remains enigmatic.

Some studies suggest that the incidences of tissue inflammation among BPH and prostate biopsy (cancerous or noncancerous) are high and are associated with proliferative inflammatory atrophy (PIA), prostatitis, prostatic intraepithelial neoplasia (PIN, low or high grade), and prostate cancer. Recently, it has been suggested that PIA occurs in association with inflammation and is considered as a precursor to prostate cancer via PIN. However, investigation of the morphological level of 1367 prostate biopsies revealed that low-grade PIN in over 90% of cases arises from normal, nonatrophic glands and is more frequently found in biopsy cores with absent or mild inflammatory burden. This concludes an inverse relationship between the prevalence of low-grade PIN and the extent of PIA lesions per patient and does not support the development of PIN from the PIA [23]. However, the diagnosis of low-grade PIN is highly subjective, and many high-grade PIN are now thought to reflect ductal spreading of otherwise invasive cancer. In fact, the majority of the published studies do not follow any specific region of the prostate or prostate lesion longitudinally, a logic that was used to indict cervical cancer intraepithelial neoplasia as a cervical cancer precursor. Another study determined the incidence of PIA in 30% of the prostate biopsies; however, the association with prostate cancer remains low and insignificant [24]. As it may be inconclusive to support PIA as a precursor of prostate cancer, the immune infiltrates and proinflammatory cytokines/chemokines eventually help the developing neoplastic cells to undergo immunoediting leading to cancer development. Therefore, the histologic inflammation alone measured by the presence of infiltrating lymphocytes in the prostate biopsy may not help to predict the risk of prostate cancer development. Even the healthy prostate tissue is found to contain immune cells infiltration [25]. The impact of proinflammatory cytokines and chemokines is equally important in facilitating tumor microenvironment contributing to prostate tumor development.

## 3. Inflammasomes as Potential Target of Prostate Inflammation

In the process of inflammation and prostate cancer development, the role of NF*κ*B has been appreciated. The nuclear factor NF*κ*B is associated with the upregulation of tumor promoting cytokines such as IL-6 and TNF-*α* [26, 27]. However, NF*κ*B is a ubiquitous transcription factor and poses a challenge as a potential target to control inflammation-associated tumor growth studies. To determine a potential target of inflammation, a number of studies have proposed the role of inflammatory cytokines and chemokines in prostate cancer [28–31]. Clinically, the measurement of IL-1*β*, IL-18, IL-6, and MIC-1/GDF-15 correlates with the risk of carcinoma and the prognosis of established cancer. While the role of IL-6 in prostate cancer is well documented, studies on serum IL-18 for its diagnostic utility in cancer are limited. Study on a cohort of 149 patients demonstrated that serum IL-18 level was significantly higher in locally advanced prostate cancer as compared to healthy control and BPH [32]. Expression of IL-18 binding protein (IL-18 BP) was significantly upregulated in patients with large volume disease, and a high serum level of IL-18 BP was correlated with Gleason score [33]. Similarly, MIC-1/GDF15 is regulated by the inflammatory cytokines and clinical evidence supports the notion that serum MIC-1/GDF-15, in combination with PSA, might improve the specificity for prostate cancer detection [34, 35]. It has been suggested that IL-1*β* and IL-18 exert immunosuppressive effects and support tumor promoting microenvironment [36, 37]. Thus, identifying the regulators of inflammation-associated cytokine/chemokines as a molecular target of cancer progression may provide an opportunity to further understand the role of inflammation in prostate cancer.

Recently, an emerging class of inflammasomes is considered as master regulators of inflammation. Inflammasomes are a group of multimeric proteins that consist of NLR protein, an apoptosis-associated speck-like protein containing a carboxyterminal CARD (ASC), and procaspase-1 [38, 39]. Assembly of inflammasomes complex activates caspase-1 leading to the secretion and the maturation of proinflammatory cytokines IL-1*β* and IL-18, which causes a wide variety of biological effects associated with infection, inflammation, and other disease processes [40–42]. Additionally, cancer cells at the advanced stage spontaneously secrete IL-1*β* where it can intrinsically activate pro-IL-1*β* and catalytic function of caspase-1 [43]. Based on caspase-1 activation, four inflammasomes including NLRP1, NLRP3, NLRC4, and the DNA sensor AIM2 (absent in melanoma) have been described [4, 38]. NLRP3 is one of the most studied inflammasomes and senses pathogens and danger signals in response to injury or infection [44, 45]. As known, various stimuli such as urine reflux, uric acid crystals, bacteria, or fungi are common elements causing injury or infection within the prostate. Therefore, such stimuli may lead to activation of inflammasome-medicated proinflammatory cytokines in the prostate driving tumor development. Linked with the prostatic inflammation in a rat model, an increased association of inflammasome proteins suggests that the activation of inflammasome complex may yield inflammatory state similar to BPH in human [46, 47]. This study showed that chemically induced chronic inflammation using carrageenan suspension leads to increase in IL-1*β* and caspase-1 proteins in association with increased NRLP1 in the prostate. Following treatment with chlorogenic acid (Chinese herbal product), there was a significant decrease both in NLRP1 and in IL-1*β* protein suggesting inflammasome mediated regulation of inflammation. It is also reported that AIM2 inflammasome plays a fundamental role in the development of human prostatic diseases [48]. AIM2 is an interferon (IFN) inducible protein, which is constitutively downregulated in prostate cancer. IFNs treated prostate cancer cell lines showed increased AIM2 activating inflammasome complex leading to production of IL-1*β* and IL-18. Also, IL-1*β* supports the skeletal colonization and metastatic progression of prostate cancer cells [49, 50]. Indeed, IL-1 is a key regulator of inflammation, and targeting IL-1 has been a major goal to modulate pathophysiology of various diseases related to autoimmunity and autoinflammatory symptoms. There are three main agents targeting IL-1: anakinra, canakinumab, and rilonacept; however, anakinra, an IL-1 receptor antagonist, which blocks the activity of both IL-1*α* and IL-1*β*, is the most widely used agent in clinics [51]. In addition to IL-1 targeting, anakinra reduces the level of IL-6, one of the most common cytokines associated with the progression and metastasis of prostate cancer. For specific targeting of IL-1*α*, a monoclonal antibody MABp1 has been tested in several phase I and II clinical studies. MABp1 targets and binds to IL-1*α*, preventing IL-1*α*-mediated tumor growth, metastasis, and invasiveness. In an open label, dose escalation phase I study, MABp1 in patients with metastatic disease of different tumor types showed no-dose limiting toxicities and was well tolerated with observed disease control [52]. Antibody MABp1 demonstrated positive results for patients with advanced symptomatic colorectal cancer in a phase 3, double-blind, placebo-controlled study [53]. Since cleavage of mature IL-1*β* is facilitated by the active caspase-1, it is very likely that, by targeting inflammasome complex, the role of inflammation-associated events can be manipulated in the prostate tumor microenvironment.

Although the role of inflammasomes in anticancer activity is not fully understood, it is suggested that the mice lacking NLRP3 have a low tumor burden and suppression of tumor metastasis [54]. NLRP3 deficiency induces NK cell infiltration and is associated with increased production of CCL5 and CXCL9 chemokines. Mice fed with high oxalate diet exhibit renal failure due to increased NLRP3-mediated inflammation [55]. However, studies in chemically induced colitis model showed that the mice lacking NLRP3 are highly susceptible to tumor formation with severe inflammation [56]. The importance of inflammasomes has been reviewed in reference to various diseases including carcinogenesis and antitumor activities with conflicting observations [57–61]. As we are progressing in this area of inflammasome research with respect to cancer studies, it is likely that the role of inflammasomes may be disease-specific. Therefore, to examine the contribution of inflammasomes to regulating prostate inflammation, it is necessary to characterize and identify the role of inflammasomes complex for appropriate targeting to modulate prostate tumor microenvironment.

## 4. Perspective

Inflammasome-associated studies in prostate cancer remain uncharacterized and are at the beginning stage. Regardless of their divergent roles in tumor growth studies, it is evident that the inflammasomes regulate inflammatory cytokines, thereby modulating the course of inflammation. Since the proinflammatory cytokines may serve as fuel for the developing neoplastic cells in the prostate, the tumor promoting effect of proinflammatory cytokines/chemokines may be reduced significantly by understanding the mechanistic regulation of inflammasomes. Keeping in view the importance of inflammation in prostate cancer, investigating the role of inflammasomes may open a new direction for therapy to target the intricacies of inflammatory pathways.
